# Supplementary material for: Half-Heusler-like compounds with wide continuous compositions and tunable p- to n-type semiconducting thermoelectrics
Source: Nat Commun. 2022 Jan 10;13:35. doi: 10.1038/s41467-021-27795-3 (PMC8748599; doi:10.1038/s41467-021-27795-3)
Supplement: Supplementary file 1 — Supplementary Information [file 41467_2021_27795_MOESM1_ESM.pdf]

Supplementary Materials for

Half-Heusler-like compounds with wide continuous compositions and tunable p- to n-type semiconducting thermoelectrics

Zirui Dong[1], Jun Luo[1,2*], Chenyang Wang[1], Ying Jiang[2], Shihua Tan[3], Yubo Zhang[3,4], Yuri Grin[5], Zhiyang Yu[6], Kai Guo[1], Jiye Zhang[1], Wenqing Zhang[3,4*]

[1] School of Materials Science and Engineering, Shanghai University, Shanghai 200444, China

[2] Materials Genome Institute, Shanghai University, Shanghai 200444, China

[3] Department of Physics and Shenzhen Institute for Quantum Science and Engineering, Southern University of Science and Technology, Shenzhen 518055, China

[4] Guangdong Provincial Key Lab for Computational Science and Materials Design, and Shenzhen Municipal Key-Lab for Advanced Quantum Materials and Devices, Southern University of Science and Technology, Shenzhen 518055, China

[5] Max-Planck-Institut für Chemische Physik fester Stoffe, Nöthnitzer Straße 40, 01187 Dresden, Germany

[6] State Key Laboratory of Photocatalysis on Energy and Environment, College of Chemistry, Fuzhou University, Fuzhou 350002, China

* Corresponding authors. E-mails: junluo@shu.edu.cn (J.L.); zhangwq@sustech.edu.cn (W.Z.)

Supplementary section 1

Calculation of the lattice thermal conductivity

The lattice thermal conductivity κ_L is normally obtained by subtracting the electronic thermal conductivity κ_e from the total thermal conductivity κ through the

Wiedemann–Franz law $\kappa_L = \kappa - \kappa_e = \kappa - L\sigma T$, where σ, L and T are the electrical conductivity, Lorenz number and absolute temperature, respectively. However, the bipolar thermal conductivity κ_b should be taken into account if intrinsic excitation presents, which gives the relationship $\kappa_L + \kappa_b = \kappa - L\sigma T$. The Lorenz number is estimated under the framework of single parabolic band model with the assumption of electron-phonon interaction. The employed equations for Lorenz number can be written as

$$S = \pm\frac{k_B}{e}\left(\frac{(r+\frac{5}{2})F_{r+\frac{3}{2}}(\eta)}{(r+\frac{3}{2})F_{r+\frac{1}{2}}(\eta)} - \eta\right) \quad \text{(S1)}$$

$$L = \left(\frac{k_B}{e}\right)^2\left(\frac{\left(r+\frac{7}{2}\right)F_{r+\frac{5}{2}}(\eta)}{\left(r+\frac{3}{2}\right)F_{r+\frac{3}{2}}(\eta)} - \left(\frac{\left(r+\frac{5}{2}\right)F_{r+\frac{3}{2}}(\eta)}{\left(r+\frac{3}{2}\right)F_{r+\frac{1}{2}}(\eta)}\right)^2\right) \quad \text{(S2)}$$

$$F(\xi) = \int_0^\infty \frac{x^i}{1+e^{x-\xi}}dx \quad \text{(S3)}$$

where S is the Seebeck coefficient, k_B the Boltzmann constant, e the free electron charge, r the scattering factor, F_i the i-th Fermi integral, η the reduced Fermi level (Fermi level over k_BT). The scattering factor r is equal to -1/2 assuming that the acoustic phonon scattering dominates the charge transport process.

Supplementary section 2

Elastic properties of $TiRu_{1+x}Sb$ at room temperature

Average sound velocity v_s can be extracted from[1,2]

$$v_s = \left(\frac{1}{3}\left(\frac{1}{v_l^3} + \frac{2}{v_t^3}\right)\right)^{-\frac{1}{3}} \quad \text{(S4)}$$

Debye temperature θ_D[1]

$$\theta_D = \frac{h}{k_B}\left(\frac{3N}{4\pi V}\right)^{\frac{1}{3}}v_s \quad \text{(S5)}$$

Bulk modulus B[3,4]

$$B = \frac{\rho(3v_l^2 - 4v_t^2)}{3} \quad \text{(S6)}$$

Gruneisen parameter γ_G[3,4]

$$\gamma_G = \frac{3}{2}\left(\frac{3v_l^2 - 4v_t^2}{v_l^2 + 2v_s^2}\right) \quad \text{(S7)}$$

Where *V* is the unit-cell volume, *N* is the number of atoms in a unit cell, *h* presents the Planck constant, and ρ is the mass density. v_l and v_t are the longitudinal and transverse sound velocities, respectively.

Supplementary section 3

Analysis of C_P

Einstein specific heat capacity

$$C_{Ein} = 3R\left(\frac{\theta_E}{T}\right)^2 \frac{e^{\theta_E/T}}{(e^{\theta_E/T} - 1)^2} \quad \text{(S8)}$$

Nuclear hyperfine specific heat capacity

$$C_{hyp}(H) = A(H)/T^2 \quad \text{(S9)}$$

Schottky specific heat capacity

$$C_{Sch} = \frac{-N\left(\frac{g_0}{g_1}\right)\left(-\frac{\varepsilon_1}{k_B T^2}\right)e^{\frac{\varepsilon_1}{k_B T}}}{\left(1 + \left(\frac{g_0}{g_1}\right)e^{\frac{\varepsilon_1}{k_B T}}\right)^2} \quad \text{(S10)}$$

Composition of C_P

$$C_P = C_{Hyp} + \gamma T + C_{Sch} + C_{Ein} + \beta T^3 \quad \text{(S11)}$$

where *R* is the Planck constant, k_B the Boltzmann constant, θ_E the Einstein temperature, *H* the magnetic field, *A*(*H*) the nuclear hyperfine specific heat capacity parameter under different magnetic fields, g_0 the degree of degeneracy with 0 energy, g_1 the degree of degeneracy with ε_1 energy, ε_1 the magnetic splitting energy level difference. γT is the specific heat capacity of electrons. βT^3 is the specific heat capacity of phonons. When the temperature is lower than $0.01\theta_D$ (usually below 5 K) C_{Dybe}=βT^3.The fitting results for the specific heat capacity are plotted in Fig. S8 and listed in Table S3.

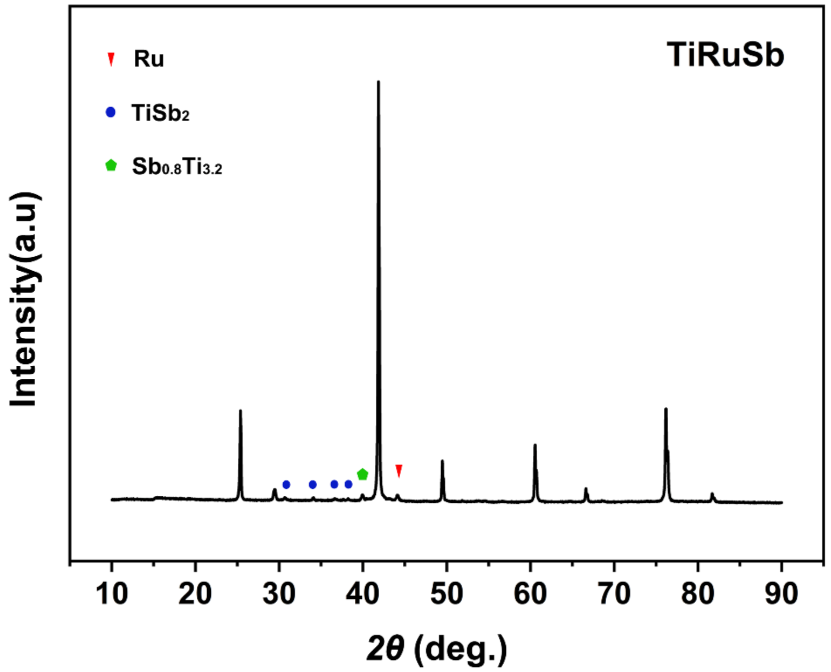

Fig. S1. Room temperature XRD pattern of TiRuSb.

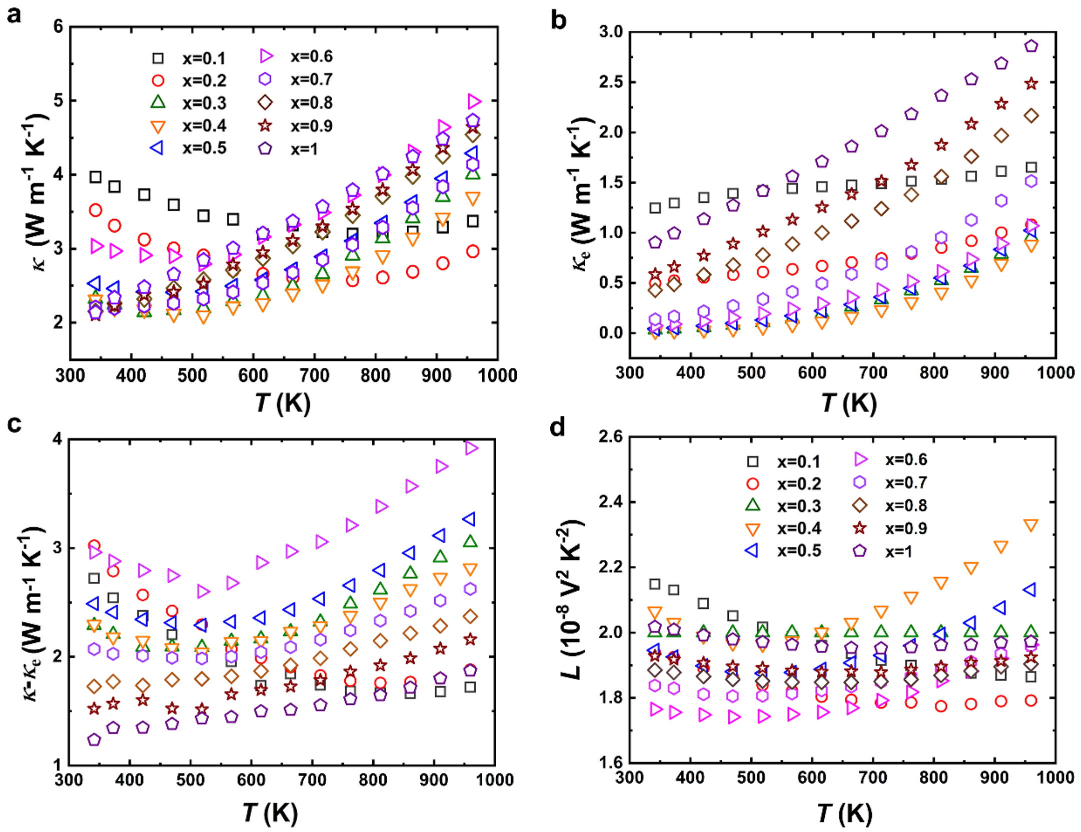

Fig. S2. Temperature-dependent thermal transport properties of $TiRu_{1+x}Sb$ samples. a, Total thermal conductivity (κ). **b,** Electronic thermal coundctivity (κ_e). **c,** κ -κ_e. **d,** Lorenz number (L).

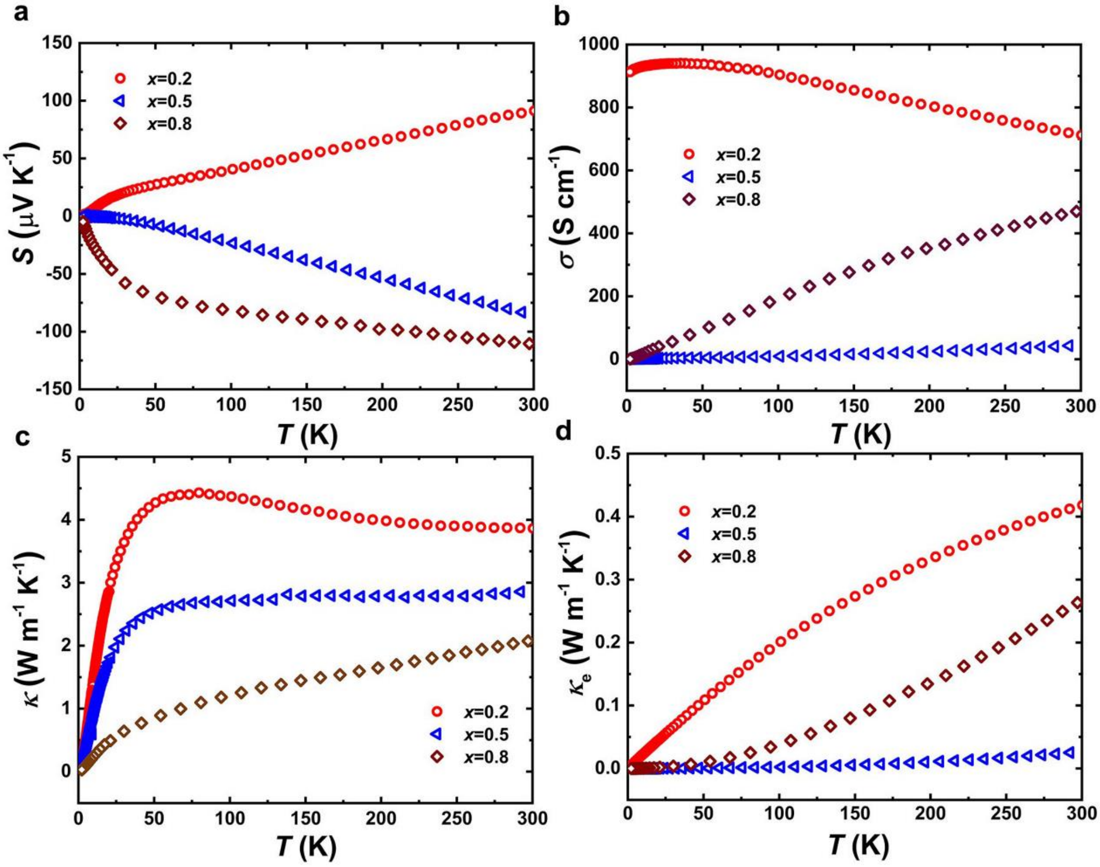

Fig. S3. Low-temperature thermoelectric transport properties of $TiRu_{1+x}Sb$ samples. a, Seebeck coefficient (*S*). **b,** Electrical conductivity (σ). **c,** Total thermal conductivity (κ). **c,** Electronic thermal conductivity (κ_e).

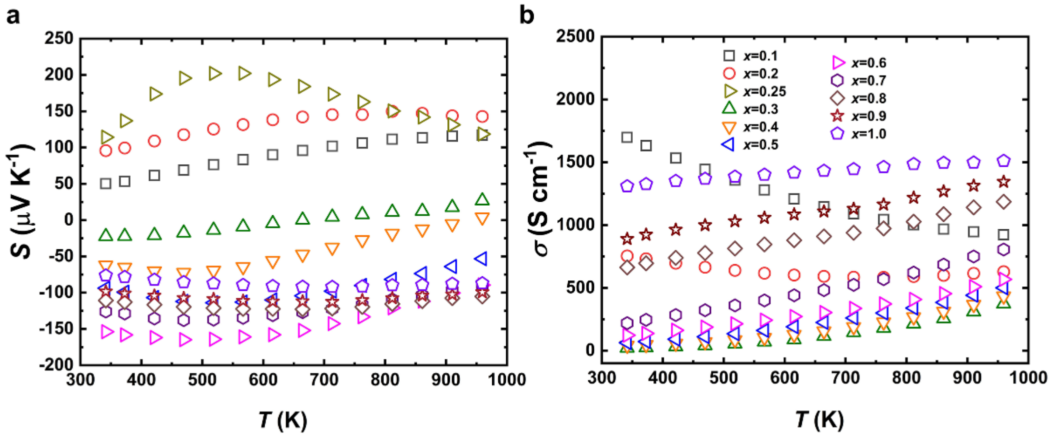

Fig. S4. Temperature-dependent electrical transport properties of $TiRu_{1+x}Sb$ samples. a, Seebeck coefficient (*S*). **b,** Electrical conductivity (σ).

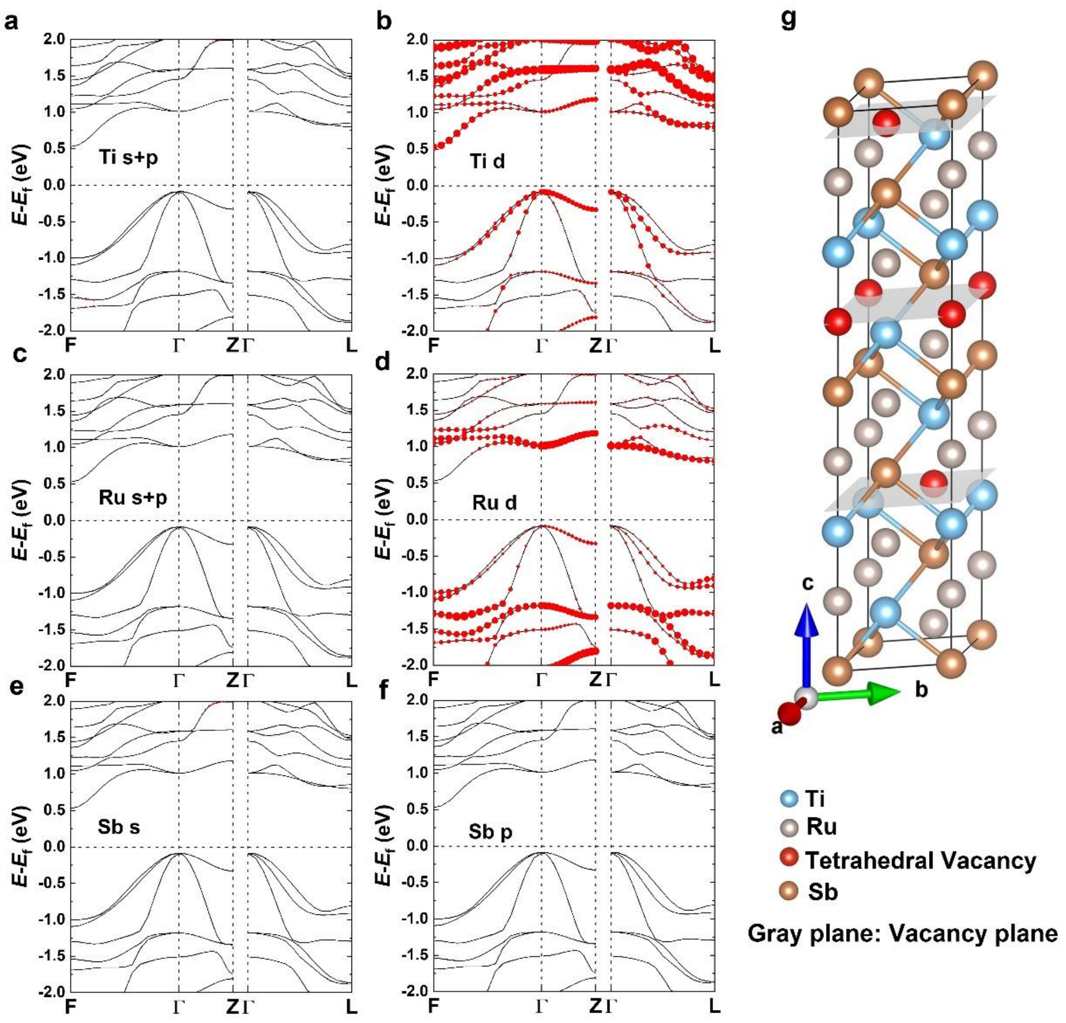

Fig. S5. Calculated electronic band structure and crystal structure of $TiRu_{1.5}Sb$. a-f, Band composition. **g,** Crystal structure. The most stable $TiRu_{1.5}Sb$ sample has a layered structure of TiRuSb and $TiRu_2Sb$ with the space group *R*3m according to our theoretical calculation.

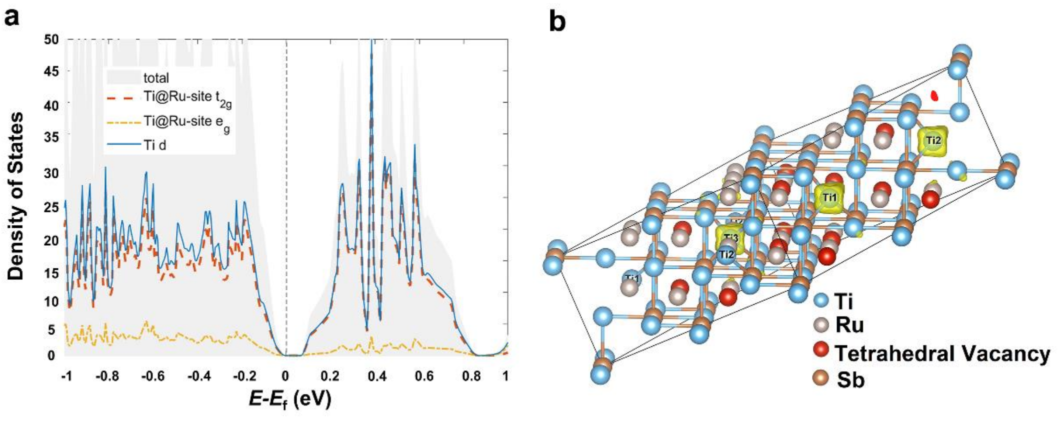

Fig. S6. Electronic structure of $Ti_{1.15}Ru_{1.2}Sb$. a, Density of states of $Ti_{1.15}Ru_{1.2}Sb$. **b,** Partial charge density of the defect states in $Ti_{1.15}Ru_{1.2}Sb$. The Ti atoms occupying the tetrahedral interstitial sites are labeled as Ti1, Ti2, and Ti3.

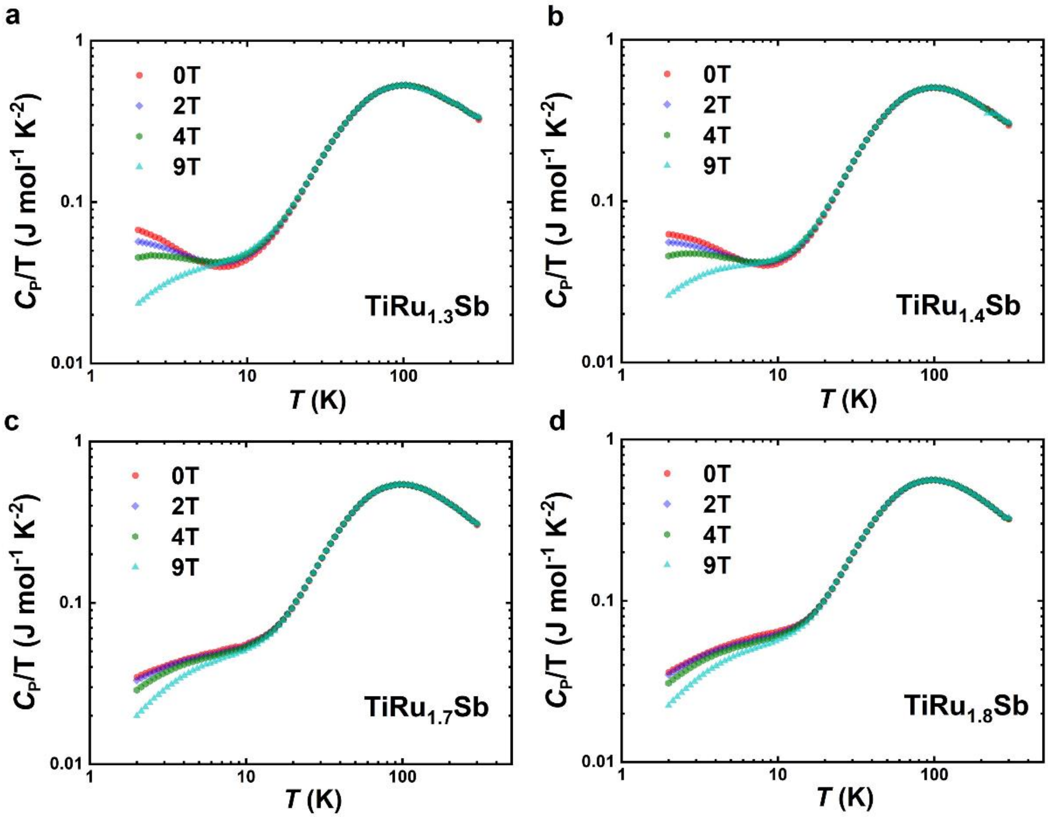

Fig. S7. Temperature-dependent specific heat capacities of TiRu$_{1+x}$Sb samples under different magnetic fields. a, TiRu$_{1.3}$Sb. **b,** TiRu$_{1.4}$Sb, **c,** TiRu$_{1.7}$Sb. **d,** TiRu$_{1.8}$Sb.

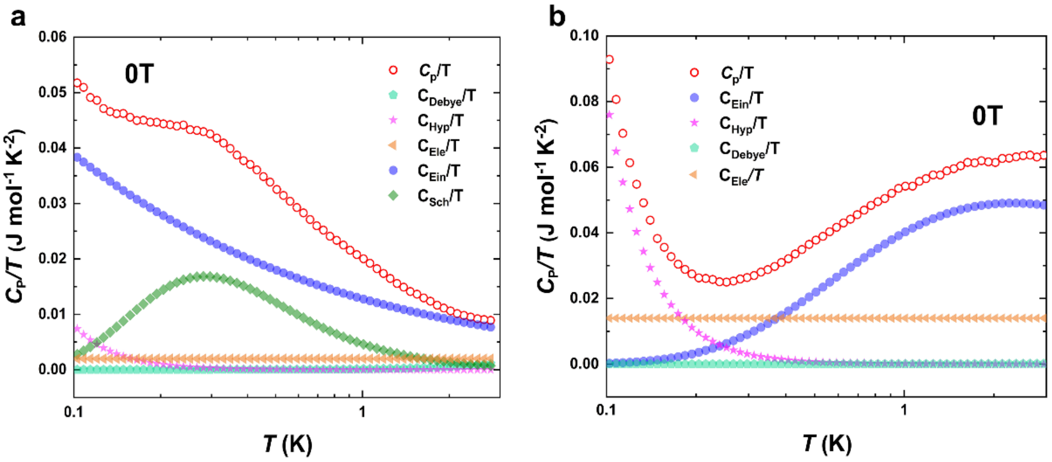

Fig. S8. Fitting results of the low-temperature specific heat capacities of TiRu$_{1+x}$Sb samples. a, TiRu$_{1.2}$Sb. **b,** TiRu$_{1.5}$Sb.

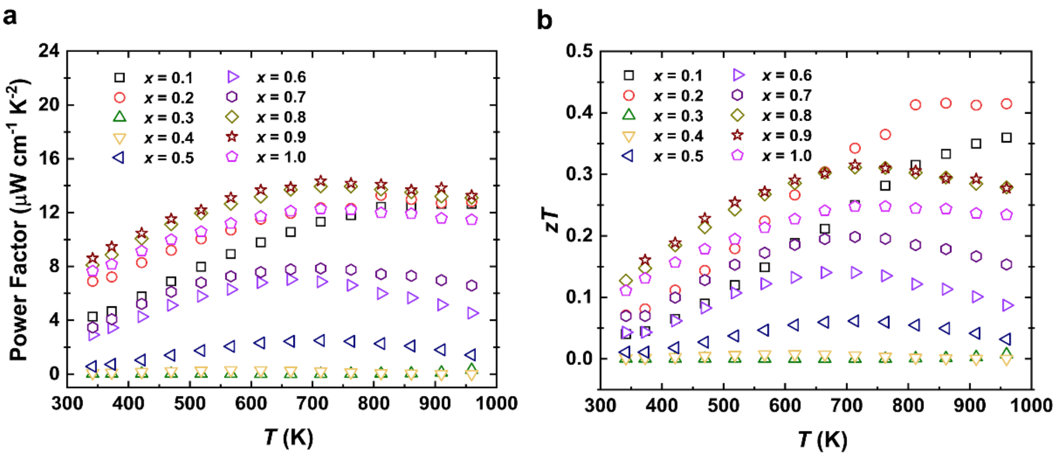

Fig. S9. Temperature-dependent thermoelectric properties of $TiRu_{1+x}Sb$ samples. a, Power factors. **b,** zT values.

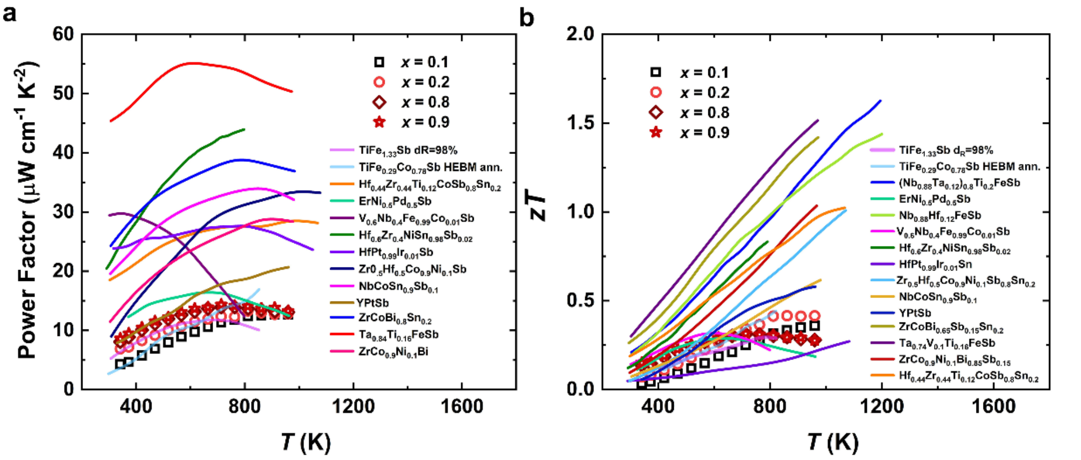

Fig. S10. Thermoelectric properties of $TiRu_{1+x}Sb$. a, Thermoelectric power factor. **b**. zT values. Typical HH [5-19] compounds are included for comparison.

Table S1. Measured compositions of $TiRu_{1+x}Sb$ samples by EMPA.

Nominal component	Ti (at. %)	Ru (at. %)	Sb (at. %)	Measured composition
$TiRu_{1.2}Sb$	32.224	36.943	30.833	$Ti_{1.045}Ru_{1.198}Sb$
$TiRu_{1.3}Sb$	31.495	38.810	29.695	$Ti_{1.061}Ru_{1.306}Sb$
$TiRu_{1.4}Sb$	31.453	38.616	28.931	$Ti_{1.087}Ru_{1.335}Sb$

Table S2. Mass densities of the $TiRu_{1+x}Sb$ samples.

Ru content	Measured density (g cm^{-3})	Theoretical density (g cm^{-3})	Relative density (%)
$x = 0.1$	8.26	8.13	*
$x = 0.2$	8.32	8.41	98.92
$x = 0.3$	8.56	8.64	99.07
$x = 0.4$	8.79	8.90	98.76
$x = 0.5$	8.94	9.13	97.92
$x = 0.6$	9.13	9.40	97.13
$x = 0.7$	9.42	9.66	97.52
$x = 0.8$	9.53	9.89	96.36
$x = 0.9$	9.65	10.15	95.07
$x = 1.0$	9.84	10.38	94.79

* The measured mass density of the sample with $x = 0.1$ is higher than its theoretical mass density because it is a multi-phase sample.

Table S3. Measured sound velocities and other estimated physical parameters at room temperature for $TiRu_{1+x}Sb$.

Ru content	v_l (m s^{-1})	v_t (m s^{-1})	v_s (m s^{-1})	θ_D (K)	B (GPa)	γ_G
$x = 0.1$	5275	3103	3439	386.8	136.3	1.43
$x = 0.2$	5921	3088	3455	391.6	203.3	1.86
$x = 0.3$	5848	3118	3483	398.4	193.2	1.78
$x = 0.4$	5952	3231	3604	415.8	195.7	1.71
$x = 0.5$	5883	3200	3569	415.2	190.7	1.71
$x = 0.6$	5866	3120	3486	408.7	195.0	1.78
$x = 0.7$	5266	2994	3328	393.2	143.5	1.55
$x = 0.8$	5784	2837	3186	379.4	206.7	2.06
$x = 0.9$	5550	2830	3170	380.7	183.1	1.93
$x = 1.0$	5184	2285	2579	311.9	181.2	2.40

Table S4. Calculated VBM and CBM effective masses of the $TiRu_{1+x}Sb$ samples.

Sample	VBM m^* (m_0)	CBM m^* (m_0)
$Ti_{1.15}Ru_{1.2}Sb$	4.45	6.62
$Ti_{1.08}Ru_{1.33}Sb$	3.80	6.44
$TiRu_{1.5}Sb$	2.05	1.80
$Ti_{0.95}Ru_{1.6}Sb$	2.19	1.89
$Ti_{0.958}Ru_{1.67}Sb$	2.05	1.80

Table S5. Fitting parameters of low-temperature specific heat capacities for $TiRu_{1+x}Sb$.

	A (mJ mol^{-1} K)	γ (mJ mol^{-1} K^{-2})	θ_E (K)	g_0/g_1	ε_1 (J)	β (mJ mol^{-1} K^{-4})
$TiRu_{1.2}Sb$	0.07	2.11	3.92	2	0.64 $\boldsymbol{k}_B$	0.89
$TiRu_{1.5}Sb$	0.08	14.12	0.24	/	/	0.81

References

1. Kurosaki, K., Kosuga, A., Muta, H., Uno, M., Yamanaka, S. Ag_9TlTe_5: A high-performance thermoelectric bulk material with extremely low thermal conductivity. *Appl. Phys. Lett.* **87**, 061919 (2005).
2. Sanditov, D. S., Belomestnykh, V. N. Relation between the parameters of the elasticity theory and averaged bulk modulus of solids. *Tech. Phys+*. **56**, 1619-1623 (2011).
3. Kong, B. *et al*. Structural, mechanical, thermodynamics properties and phase transition of FeVSb. *Physica B.* **406**, 3003-3010 (2011).
4. Pei, Y. L. *et al*. High thermoelectric performance of oxyselenides: intrinsically low thermal conductivity of Ca-doped BiCuSeO. *NPG Asia Mater.* **5**, e47 (2013).
5. Tavassoli, A. *et al*. The half -Heusler system $Ti_{1+x}Fe_{1.33-x}Sb$-TiCoSb with Sb/Sn substitution: phase relations, crystal structures and thermoelectric properties. *Dalton. Trans.* **47**, 879-897 (2018).
6. Yan, X. *et al*. Thermoelectric Property Study of Nanostructured p-Type Half-Heuslers (Hf, Zr, Ti)$CoSb_{0.8}Sn_{0.2}$. *Adv. Energy Mater.* **3**, 1195-1120 (2013).
7. Yu, J. *et al*. Unique Role of Refractory Ta Alloying in Enhancing the Figure of Merit of NbFeSb Thermoelectric Materials. *Adv. Energy Mater.* **8**, 1701313 (2018).
8. Kawano, K., Kurosaki, K., Muta, H., Yamanaka, S. Substitution effect on the thermoelectric properties of p-type half-Heusler compounds: $ErNi_{1-x}Pd_xSb$. *J. Appl. Phys.* **104**, 013714 (2008).
9. Fu, C. G. *et al*. Realizing high figure of merit in heavy-band p-type half-Heusler thermoelectric materials. *Nat. Commun.* **6**, 8144 (2015).
10. Fu, C. G. *et al*. Electron and phonon transport in Co-doped $FeV_{0.6}Nb_{0.4}Sb$ half-Heusler thermoelectric materials. *J. Appl. Phys.* **114**, 134905 (2013).
11. Yu, C. *et al*. High-performance half-Heusler thermoelectric materials $Hf_{1-x}Zr_xNiSn_{1-y}Sb_y$ prepared by levitation melting and spark plasma sintering. *Acta Mater.* **57**, 2757-2764 (2009).
12. Kimura, Y., Zama, A. Thermoelectric properties of p-type half-Heusler compound HfPtSn and improvement for high-performance by Ir and Co additions. *Appl. Phys. Lett.* **89**, 172110 (2006).
13. He, R. *et al*. Improved thermoelectric performance of n-type half-Heusler $MCo_{1-x}Ni_xSb$ (M = Hf, Zr). *Mater. Today Phys.* **1**, 24-30 (2017).
14. He, R. *et al*. Enhanced thermoelectric properties of n-type NbCoSn half-Heusler by improving phase purity. *APL Materials*. **4**, 104804 (2016).
15. Li, G. H., Kurosaki, K., Ohishi, Y., Muta, H., Yamanaka, S. High Temperature Thermoelectric Properties of Half-Heusler Compound PtYSb. *JPN J APPL PHYS*. **52**, 041804 (2013).
16. Zhu, H. T. *et al*. Discovery of ZrCoBi based half Heuslers with high thermoelectric conversion efficiency. *Nat. Commun.* **9**, 2497 (2018).
17. Zhu, H. T. *et al*. Discovery of TaFeSb-based half-Heuslers with high thermoelectric performance. *Nat. Commun.* **10**, 270 (2019).

18. Zhu, H. T. *et al*. Understanding the asymmetrical thermoelectric performance for discovering promising thermoelectric materials. *Sci. Adv.* **6**, eaav5813 (2019).
19. Mao, J. *et al*. Thermoelectric Properties of n-type ZrNiPb-Based Half-Heuslers. *Chem. Mater.* **29**, 867-872 (2017).
