# Supplementary material for: Half-Heusler-like compounds with wide continuous compositions and tunable p- to n-type semiconducting thermoelectrics
Source: Nat Commun. 2022 Jan 10;13:35. doi: 10.1038/s41467-021-27795-3 (PMC8748599; doi:10.1038/s41467-021-27795-3)
Supplement: Supplementary file 4 — Lasing Reporting Summary [file 41467_2021_27795_MOESM4_ESM.pdf]

nature research

Corresponding author(s): Jun Luo

Lasing Reporting Summary

Nature Research wishes to improve the reproducibility of the work that we publish. This form is intended for publication with all accepted papers reporting claims of lasing and provides structure for consistency and transparency in reporting. Some list items might not apply to an individual manuscript, but all fields must be completed for clarity.

For further information on Nature Research policies, including our data availability policy, see Authors & Referees.

Experimental design

Please check: are the following details reported in the manuscript?

1. Threshold

Plots of device output power versus pump power over a wide range of values indicating a clear threshold

☐ Yes ☒ No

In this work, the only laser involved is the measurement of thermal conductivity. In the laser thermal conductivity instrument, the laser component is only used as a heat source and has no any other purpose. Therefore, this work does not contain any detailed description of the laser.

2. Linewidth narrowing

Plots of spectral power density for the emission at pump powers below, around, and above the lasing threshold, indicating a clear linewidth narrowing at threshold

☐ Yes ☒ No

In this work, the only laser involved is the measurement of thermal conductivity. In the laser thermal conductivity instrument, the laser component is only used as a heat source and has no any other purpose. Therefore, this work does not contain any detailed description of the laser.

Resolution of the spectrometer used to make spectral measurements

☐ Yes ☒ No

In this work, the only laser involved is the measurement of thermal conductivity. In the laser thermal conductivity instrument, the laser component is only used as a heat source and has no any other purpose. Therefore, this work does not contain any detailed description of the laser.

3. Coherent emission

Measurements of the coherence and/or polarization of the emission

☐ Yes ☒ No

In this work, the only laser involved is the measurement of thermal conductivity. In the laser thermal conductivity instrument, the laser component is only used as a heat source and has no any other purpose. Therefore, this work does not contain any detailed description of the laser.

4. Beam spatial profile

Image and/or measurement of the spatial shape and profile of the emission, showing a well-defined beam above threshold

☐ Yes ☒ No

In this work, the only laser involved is the measurement of thermal conductivity. In the laser thermal conductivity instrument, the laser component is only used as a heat source and has no any other purpose. Therefore, this work does not contain any detailed description of the laser.

5. Operating conditions

Description of the laser and pumping conditions
Continuous-wave, pulsed, temperature of operation

☐ Yes ☒ No

In this work, the only laser involved is the measurement of thermal conductivity. In the laser thermal conductivity instrument, the laser component is only used as a heat source and has no any other purpose. Therefore, this work does not contain any detailed description of the laser.

Threshold values provided as density values (e.g. W cm^{-2} or J cm^{-2}) taking into account the area of the device

☐ Yes ☒ No

In this work, the only laser involved is the measurement of thermal conductivity. In the laser thermal conductivity instrument, the laser component is only used as a heat source and has no any other purpose. Therefore, this work does not contain any detailed description of the laser.

6. Alternative explanations

Reasoning as to why alternative explanations have been ruled out as responsible for the emission characteristics
e.g. amplified spontaneous, directional scattering; modification of fluorescence spectrum by the cavity

☐ Yes ☒ No

In this work, the only laser involved is the measurement of thermal conductivity. In the laser thermal conductivity instrument, the laser component is only used as a heat source and has no any other purpose. Therefore, this work does not contain any detailed description of the laser.

7. Theoretical analysis

Theoretical analysis that ensures that the experimental values measured are realistic and reasonable
e.g. laser threshold, linewidth, cavity gain-loss, efficiency

☐ Yes ☒ No

In this work, the only laser involved is the measurement of thermal conductivity. In the laser thermal conductivity instrument, the laser component is only used as a heat source and has no any other purpose. Therefore, this work does not contain any detailed description of the laser.

8. Statistics

Number of devices fabricated and tested

☐ Yes
☒ No

In this work, the only laser involved is the measurement of thermal conductivity. In the laser thermal conductivity instrument, the laser component is only used as a heat source and has no any other purpose. Therefore, this work does not contain any detailed description of the laser.

Statistical analysis of the device performance and lifetime (time to failure)

☐ Yes
☒ No

In this work, the only laser involved is the measurement of thermal conductivity. In the laser thermal conductivity instrument, the laser component is only used as a heat source and has no any other purpose. Therefore, this work does not contain any detailed description of the laser.
